# NEAT1_2 functions as a competing endogenous RNA to regulate ATAD2 expression by sponging microRNA-106b-5p in papillary thyroid cancer

**DOI:** 10.1038/s41419-018-0418-z

**Published:** 2018-03-07

**Authors:** Wei Sun, Xiabin Lan, Hao Zhang, Zhihong Wang, Wenwu Dong, Liang He, Ting Zhang, Ping Zhang, Jinhao Liu, Yuan Qin

**Affiliations:** 1 Department of Thyroid Surgery, The First Hospital of China Medical University, Shenyang, Liaoning Province China; 2 Department of Head and Neck Surgery, Zhejiang Province Cancer Hospital, Hangzhou, Zhejiang Province China

## Abstract

Nuclear paraspeckle assembly transcript 1 (NEAT1), a long non-coding RNA (lncRNA), is a core structural component of paraspeckles and is essential for paraspeckle formation. NEAT1 comprises two different isoforms: NEAT1_1 (3.7 kb) and NEAT1_2 (23 kb). Recently, NEAT1 has been shown to have oncogenic roles and to facilitate tumorigenesis in various human cancers. However, the function of NEAT1 in papillary thyroid cancer (PTC) is not well understood. The relative expression levels of NEAT1_2, ATPase family AAA domain-containing protein 2 (ATAD2), and microRNA-106b-5p (miR-106b-5p) were assessed via quantitative real-time reverse transcription polymerase chain reaction (qRT-PCR). Four PTC cell lines were used to detect the relative expression of NEAT1_2. The effects of NEAT1_2 on PTC cells were studied by RNA interference approaches in vitro. The effects of NEAT1_2 on downstream proteins were detected by western blotting. The underlying mechanism was clarified by a rescue experiment, and three dual-luciferase reporter assays. NEAT1_2 expression was markedly increased in PTC tissues and the PTC cell lines (K1 and TPC1). The relative expression level of NEAT1_2 was positively associated with TNM stage and tumor size. NEAT1_2 knockdown led to a significant inhibition of growth and metastasis, and induced apoptosis in PTC cells. Knockdown of NEAT1_2 significantly inhibited malignant biological behavior by downregulating the oncogene ATAD2. In addition, NEAT1_2 could act as a competing endogenous RNA to regulate the expression of ATAD2 through downregulating miR-106b-5p. Taken together, our results indicated that NEAT1_2 is overexpressed in PTC. NEAT1_2 could function as a competing endogenous RNA to regulate ATAD2 expression by sponging miR-106b-5p in PTC. Targeting NEAT1_2 could be a promising therapeutic strategy for patients with PTC.

## Background

Thyroid cancer is the most common malignancy of the endocrine system, accounting for 5–10% of all malignancies in women. Of all the various histological subtypes, papillary thyroid carcinoma (PTC) is the most common histotype, accounting for 85–90% of all cases^1,2^. The incidence of PTC has steadily increased over the past 40 years. Most PTCs are effectively treated by surgical removal, followed by adjuvant radioactive iodine (RAI) therapy, and the 5-year survival rate is over 95%^3^. However, some patients do not respond to RAI therapy or progress to metastatic disease. In these cases, prognosis is poor and the 10-year survival rate drops to 40%^4^. It was also reported that 10–15% of patients with PTC exhibit relapse and metastasis after therapy^3,5^. Thus, it is urgent to identify potential biomarkers and therapeutic targets that correlate with tumorigenesis and progression in PTC.

Long non-coding RNAs (lncRNAs) constitute a newly identified class of RNAs that are more than 200 nucleotides in length and regulate gene expression through the control of transcription or post-transcription, epigenetic modification, and mRNA splicing^6^. Recent studies indicated that lncRNAs are involved in various biological processes and diseases in humans^7–9^. However, few studies have been conducted on the effects of lncRNAs in PTC. An lncRNA termed nuclear paraspeckle assembly transcript 1 (NEAT1), which was first identified in patients with multiple endocrine neoplasia and is located on chromosome 11q13.1, is an important component of nuclear paraspeckles. It has two different isoforms: NEAT1_1 (3.7 kb) and NEAT1_2 (23 kb)^10,11^. Recent reports have suggested that NEAT1 contributes to tumorigenesis in various cancers, such as lung cancer, prostate cancer, hepatocellular cancer, laryngeal squamous cell cancer, and gastric cancer^12–16^. Our previous lncRNA expression profile microarray study in PTC showed that NEAT1_2 expression was significantly upregulated in PTC compared with that in non-cancerous tissues. The fold change was 4.65 in our genome-wide analysis^17^.

Research has shown that lncRNAs might function as a competing endogenous RNAs (ceRNAs, or a molecular sponge) to modulate microRNAs (miRNAs). As an oncogene, NEAT1 could inhibit miR-449-5p expression, resulting in upregulated c-MET expression, which promoted proliferation, migration, and invasion, and inhibited apoptosis of glioma cells^18^. Moreover, NEAT1 could regulate the miR-377-3p/E2F3 pathway in non-small cell lung cancer, and regulate the miR-204/ZEB1 axis in nasopharyngeal carcinoma^19,20^. Taken together, NEAT1 could play a critical role in human cancers by inhibiting the effects of miRNAs.

ATAD2 (ATPase family AAA domain-containing 2), which can activate transcription factors such as E2F family members, estrogen receptor, and MYC, is an epigenetic regulator whose encoding gene maps to chromosome 8q24 (refs. ^21,22^). Studies have found that it is aberrantly expressed in hepatocellular carcinoma, prostate cancer, lung cancer, ovarian cancer, and cervical cancer^23^. Furthermore, elevated ATAD2 protein levels are related to tumor stage, histological grade, and lymph node metastasis. Thus, ATAD2 might function as a biomarker for tumor proliferation and metastasis, and as a prognostic factor in many human cancers. However, the expression and function of ATAD2 in PTC remain unknown.

In this study, we investigated the relative expression of NEAT1_2 in PTC and adjacent non-cancerous tissues. We then analyzed the potential relationship between the NEAT1_2 levels and clinicopathological features. The potential functions of NEAT1_2, such as cell growth, metastasis, and apoptosis, were detected in PTC cells. Downregulation of NEAT1_2 inhibited malignant biological behaviors by regulating the expression of downstream target protein ATAD2. In addition, we found that NEAT1_2 may act as a ceRNA that regulates ATAD2 by modulating miR-106b-5p. Together, these data contribute to the characterization of the molecular mechanisms of PTC progression.

## Results

### NEAT1_2 was frequently upregulated in PTC samples and was associated with tumor size and TNM stage in PTC

**Fig. 1:**
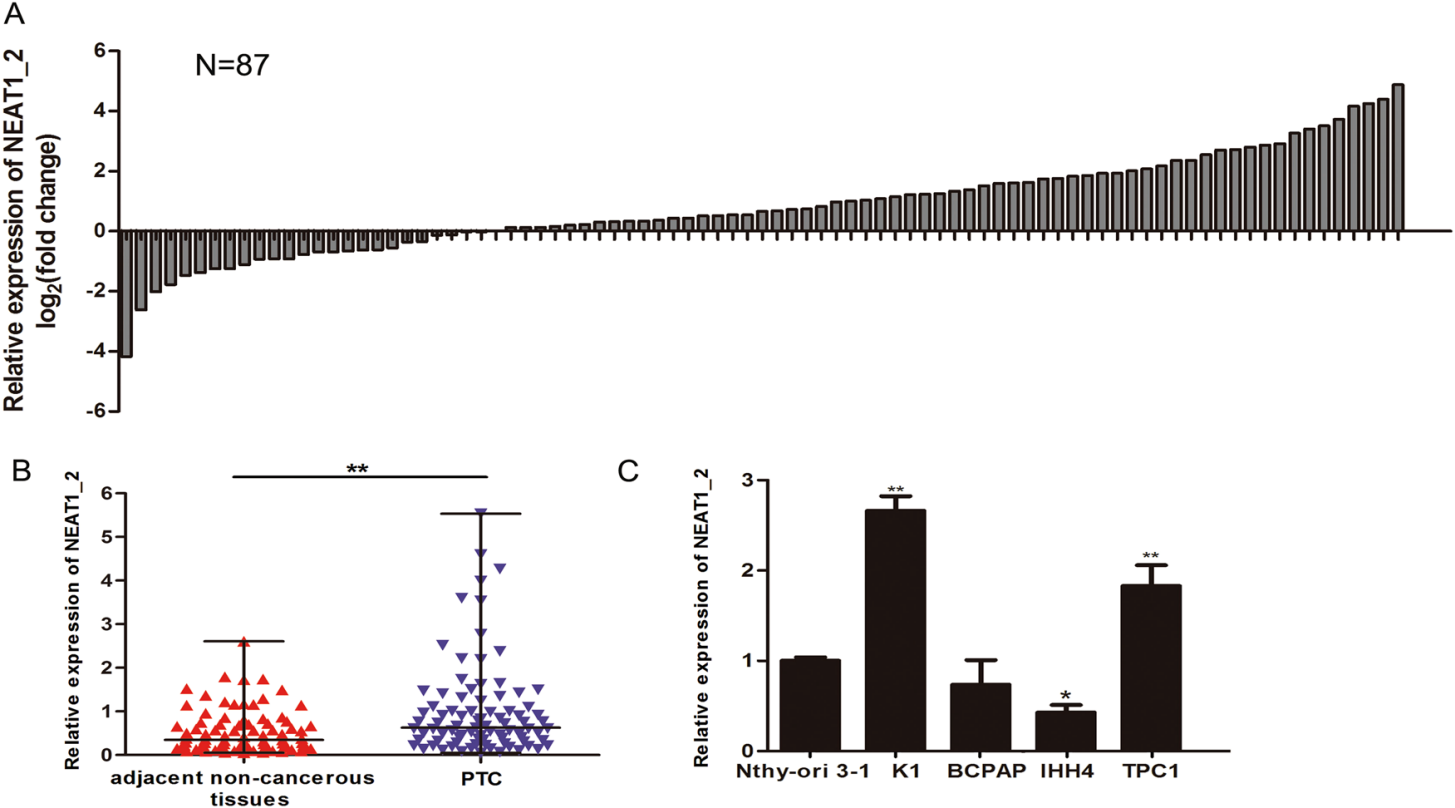
The relative expression of NEAT1_2 in PTC tissues and adjacent non-cancerous tissues, and in PTC cell lines. **a** The fold change of NEAT1_2 expression between PTC and corresponding adjacent non-cancerous tissues. **b** The relative expression levels of NEAT1_2 in 87 pairs of PTC tissues and adjacent non-cancerous tissues, as determined by qRT-PCR. The Wilcoxon signed-rank test was used to analyze the differences between the two groups; data are presented as the median with a range. **P < 0.01. **c** The relative expression levels of NEAT1_2 in PTC cell lines (K1, BCPAP, IHH4, and TPC1) and a normal human thyroid follicular epithelial cell line (Nthy-ori 3-1). Data were analyzed using independent samples *t*-test. *P < 0.05, **P < 0.01 vs. the Nthy-ori 3-1 group

The relative expression level of NEAT1_2 in 87 pairs of PTC and adjacent non-cancerous tissues was detected and analyzed by real-time reverse transcription polymerase chain reaction (qRT-PCR). The results showed that NEAT1_2 expression was significantly upregulated in human PTC tissues compared with that in adjacent non-cancerous tissues (Fig. 1a, b). The relative expression of NEAT1_2 was also detected in four PTC cell lines (K1, BCPAP, IHH4, and TPC1) and a normal human thyroid follicular epithelial cell line (Nthy-ori 3-1). NEAT1_2 expression was higher in K1 and TPC1 cell lines than in the Nthy-ori 3-1 cell line (Fig. 1c). To further explore the potential clinical significance of NEAT1_2 in PTC, we examined the correlations between the relative expression of NEAT1_2 and the clinicopathological features. The median value of lncRNA NEAT1_2 relative expression in PTC tissues was 0.6272, and this was used as a cut-off value to divide the patients into the following two groups: high NEAT1_2 relative expression (≥0.6272; *n* = 44) and low NEAT1_2 relative expression (<0.6272; *n* = 43). The clinicopathological characteristics of the 87 PTC patients are shown in Table 1 and Supplementary Figure 1. A large difference in the relative expression of NEAT1_2 in PTC tissues was positively associated with tumor size (*P* = 0.014) and TNM stage (*P* = 0.011). Based on these findings, we speculated that NEAT1_2 might play a vital role in PTC development.

**Table 1:** Correlation between NEAT1_2 expression and clinicopathological features in papillary thyroid cancer (PTC) (*n* = 87)

Characteristics	*n*	High expression (%)	Low expression (%)	*P*
Gender				
Male	29	15 (51.7)	14 (48.3)	0.879
Female	58	29 (50.0)	29 (50.0)	
Age (years)				
<45	48	21 (43.7)	27 (56.3)	0.158
≥45	39	23 (59.0)	16 (41.0)	
Extrathyroidal extension				
Yes	30	15 (50.0)	15 (50.0)	0.938
No	57	29 (50.9)	28 (49.1)	
TNM staging				
I–II	53	21 (39.6)	32 (60.4)	0.011*
III–IV	34	23 (67.6)	11 (32.4)	
Lymph node metastasis				
Yes	63	31 (49.2)	32 (50.8)	0.679
No	24	13 (54.2)	11 (45.8)	
Multicentricity				
Yes	52	29 (55.8)	23 (44.2)	0.238
No	35	15 (42.9)	20 (57.1)	
Tumor size (cm)				
<2	43	16 (37.2)	27 (62.8)	0.014*
≥2	44	28 (63.6)	16 (36.4)	

*P < 0.05

### Knockdown of NEAT1_2 inhibited growth and induced apoptosis in PTC cells

**Fig. 2:**
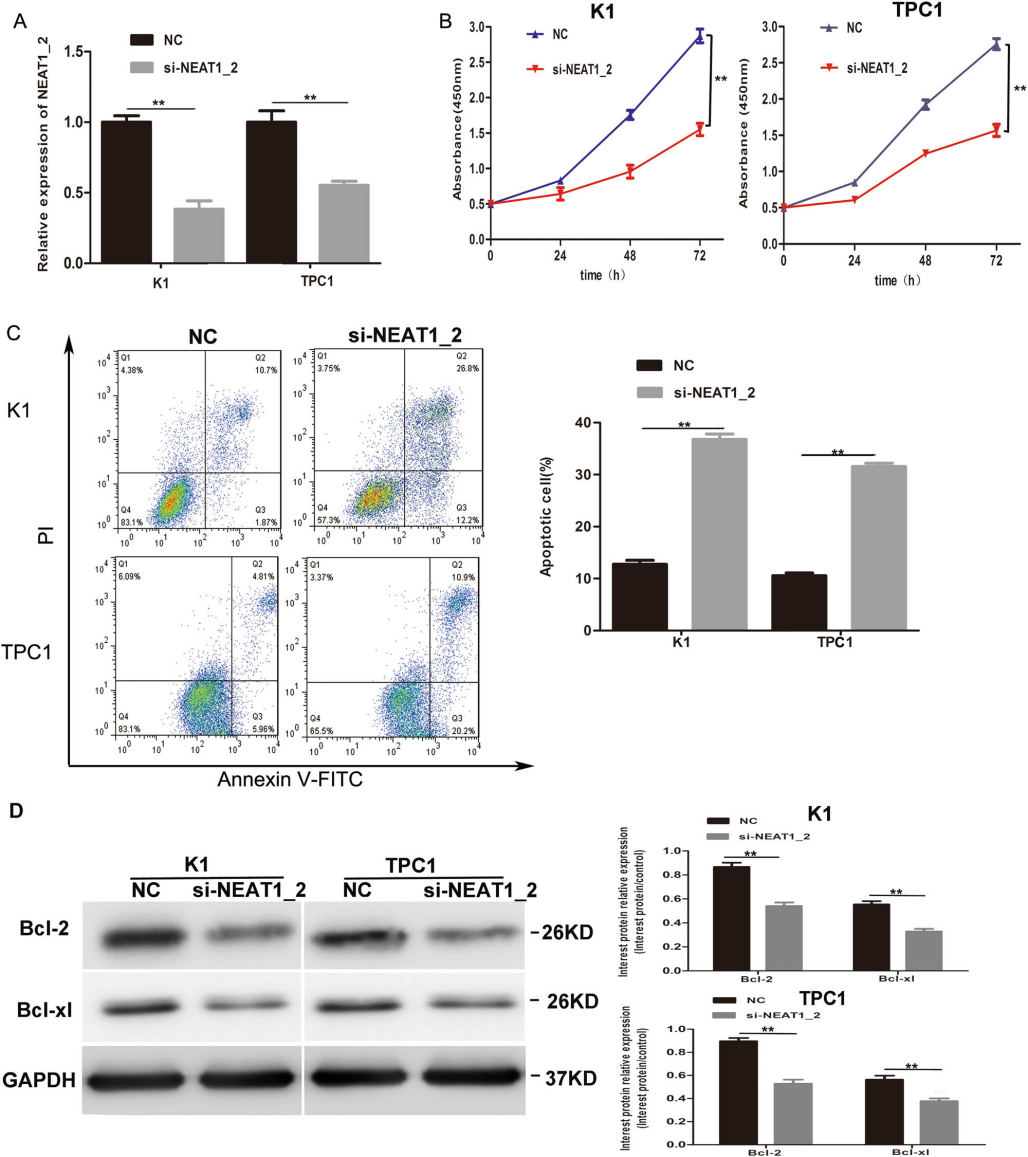
Knockdown of NEAT1_2 inhibited growth and induced apoptosis in PTC cells. **a** QRT-PCR analysis of NEAT1_2 interference efficiency after si-NEAT1_2 or NC transfection in PTC cells. **P < 0.01 vs. NC. **b** The CCK-8 assay was used to evaluate the growth after transfection with si-NEAT1_2 or NC in PTC cells. **P < 0.01 vs. NC. **c** Flow cytometry was used to evaluate the effects of apoptosis after transfection with si-NEAT1_2 or NC in PTC cells. **P < 0.01 vs. NC. **d** Apoptosis relevant proteins (Bcl-2, Bcl-xl) were detected by western blotting analysis after transfection with si-NEAT1_2 or NC in PTC cells. **P < 0.01 vs. NC. In this figure, the statistical differences were all analyzed using independent samples *t*-test. All data are presented as the mean ± S.D.

The CCK-8 assay was applied to detect roles of NEAT1_2 in cell growth. We firstly downregulated NEAT1_2 in two PTC cell lines (K1 and TPC1). The knockdown efficiency of si-NEAT1_2 in K1 and TPC1 cells is shown in Fig. 2a. The results showed that NEAT1_2 knockdown significantly inhibited cell growth of K1 and TPC1 cells (Fig. 2b). To further determine whether apoptosis was a contributing factor to cell growth inhibition, we performed flow-cytometry analysis, the results of which indicated that NEAT1_2 knockdown remarkably induced apoptosis (Fig. 2c). In addition, we found that inhibition of NEAT1_2 regulated apoptosis-associated proteins Bcl-2 and Bcl-xl (Fig. 2d). Taken together, these data suggested that NEAT1_2 knockdown suppressed PTC cell growth and induced apoptosis by regulating Bcl-2 and Bcl-xl in vitro.

### Knockdown of NEAT1_2 inhibited the migration and invasion of PTC cells

**Fig. 3:**
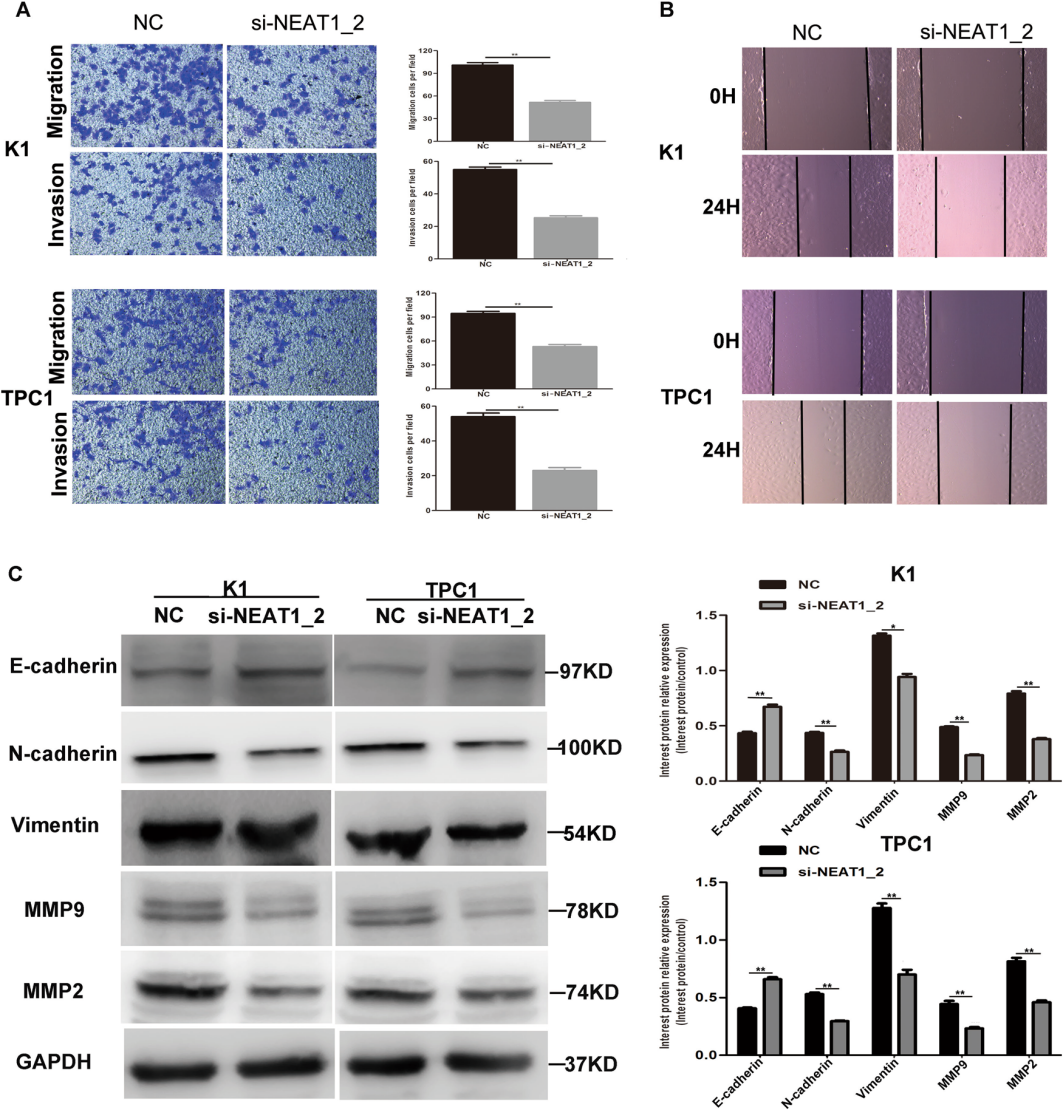
Knockdown of NEAT1_2 inhibited the migration and invasion in PTC cells. **a** Transwell assays were used to evaluate the migration and invasion in PTC cells after transfection with si-NEAT1_2 or NC. Data are presented as the mean ± S.D., analyzed using independent samples t-test. **P < 0.01 vs. NC. **b** A wound healing assay was applied to analyze the migration capacity in PTC cells after transfection with si-NEAT1_2 or NC. **c** The levels of migration and invasion related proteins (E-cadherin, Vimentin, N-cadherin, MMP9, and MMP2) were detected by western blotting analysis after transfection with si-NEAT1_2 or NC in PTC cells. Data are presented as the mean ± S.D., analyzed using independent samples *t*-test. *P < 0.05, **P < 0.01 vs. NC

Next, we evaluated cell migration and invasion using Transwell and wound healing assays. The results of the Transwell assay indicated that knockdown of NEAT1_2 impeded the migration and invasion abilities of PTC cells (Fig. 3a). Moreover, the wound healing assay confirmed the effect of NEAT1_2 knockdown on migration (Fig. 3b). To better understand the mechanisms of NEAT1_2-regulated metastasis in PTC, we detected the expression of epithelial–mesenchyme transition (EMT)-associated proteins and matrix metalloproteinases (MMPs). Western blotting analysis revealed that the epithelial marker gene E-cadherin was significantly increased in the si-NEAT1_2 group (Fig. 3c). However, the mesenchymal markers Vimentin and N-cadherin were significantly decreased (Fig. 3c). In addition, MMP9 and MMP2 were also downregulated in the si-NEAT1_2 group (Fig. 3c). These findings suggested that NEAT1_2 could influence the migration and invasion of PTC cell by regulating EMT-associated proteins and MMPs.

### Knockdown of NEAT1_2 downregulated ATAD2 in PTC cells

**Fig. 4:**
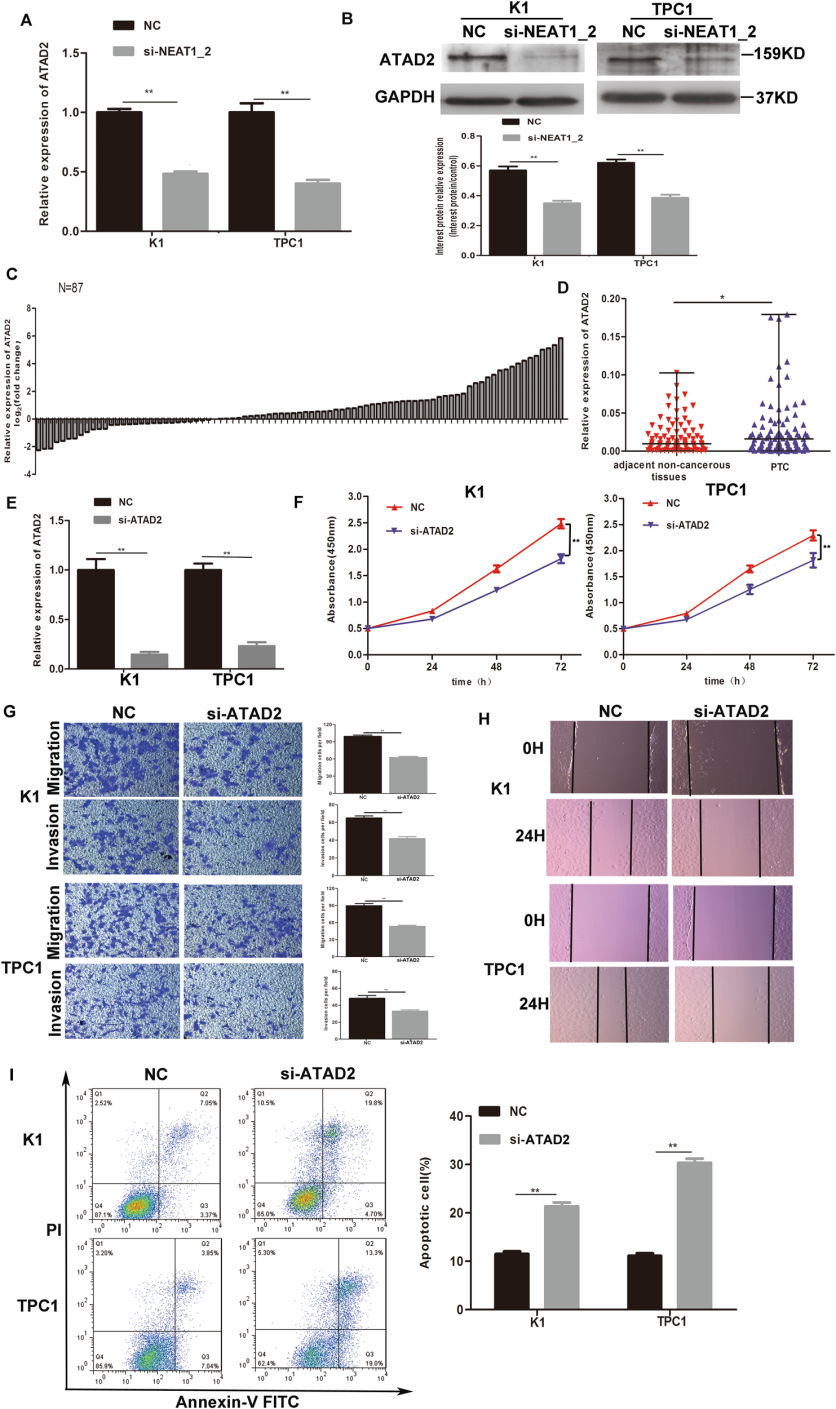
Knockdown of NEAT1_2 downregulated ATAD2 in PTC cells. **a** The relative mRNA expression of *ATAD2* was detected by qRT-PCR in PTC cells after transfection with si-NEAT1_2 or NC. Data are presented as the mean ± S.D., analyzed using independent samples *t*-test. **P < 0.01 vs. NC. **b** Western blotting was applied to detect the protein level of ATAD2 in PTC cells transfection with si-NEAT1_2 or NC. Data are presented as the mean ± S.D., analyzed using independent samples *t*-test. **P < 0.01 vs. NC. **c** The fold change in *ATAD2* expression between PTC and corresponding adjacent non-cancerous tissues. **d** The relative expression of *ATAD2* was detected in 87 pairs of PTC tissues and adjacent non-cancerous tissues by qRT-PCR. The Wilcoxon signed-rank test was used to analyze the differences between the two groups; data are presented as the median with range. *P < 0.05. **e** The knockdown efficiency of *ATAD2* was evaluated by qRT-PCR. Data are presented as the mean ± S.D., analyzed using independent samples* t*-test. **P < 0.01 vs. NC. **f** The CCK-8 assay was used to evaluate cell growth after transfection with si-ATAD2 or NC in PTC cells. Data are presented as the mean ± S.D., analyzed using independent samples *t*-test. **P < 0.01 vs. NC. **g** Transwell assay analysis was used to evaluate the migration and invasion of PTC cells after transfection with si-ATAD2 or NC. Data are presented as the mean ± S.D., analyzed using independent samples *t*-test. **P < 0.01 vs. NC. **h** A wound healing assay was applied to analyze the migration capacity of PTC cells after transfection with si-ATAD2 or NC. **i** Flow cytometry was used to evaluate the effects on apoptosis after transfection with si-ATAD2 or NC in PTC cells. Data are presented as the mean ± S.D., analyzed using independent samples t-test. **P < 0.01 vs. NC

LncRNAs are involved in tumorigenesis by regulating downstream proteins. Interestingly, we found significantly lower ATAD2 mRNA and protein levels in the si-NEAT1_2 group compared with those in the NC group (Fig. 4a, b). Thus, we speculated that the knockdown of NEAT1_2 might inhibit the cells’ malignant behavior by regulating ATAD2. To investigate the expression and functional roles of ATAD2 in PTC, the relative expression level of *ATAD2* was detected in 87 pairs of PTC and adjacent non-cancerous tissues using qRT-PCR. We found that *ATAD2* was significantly upregulated in tumor tissues compared with its expression in adjacent non-cancerous tissues (Fig. 4c, d). Examination of the correlation between *ATAD2* relative expression and clinicopathological features showed that *ATAD2* upregulation correlated with larger tumor size (Table 2 and Supplementary Figure 1) (*P* = 0.014). To examine the functional roles of ATAD2 in PTC, we downregulated *ATAD2* expression in PTC cells. The knockdown efficiency of si-ATAD2 is shown in Fig. 4e. We observed that compared with the control treatment, *ATAD2* knockdown in PTC cell led to a notable reduction in cell viability (Fig. 4f), a decreased ability for cell migration and invasion (Fig. 4g, h), and significantly induced cell apoptosis (Fig. 4i). These results indicated the possibility that NEAT1_2 could play a functional role by regulating the expression of ATAD2.

**Table 2:** Correlation between ATAD2 expression and clinicopathological features in papillary thyroid cancer (PTC) (*n* = 87)

Characteristics	*n*	High expression (%)	Low expression (%)	*P*
Gender				
Male	29	16 (55.2)	13 (44.8)	0.544
Female	58	28 (48.3)	30 (51.7)	
Age (years)				
<45	48	23 (47.9)	25 (52.1)	0.582
≥45	39	21 (53.8)	18 (46.2)	
Extrathyroidal extension				
Yes	30	19 (63.3)	11 (36.7)	0.084
No	57	25 (43.9)	32 (56.1)	
TNM staging				
I–II	53	27 (50.9)	26 (49.1)	0.932
III–IV	34	17 (50.0)	17 (50.0)	
Lymph node metastasis				
Yes	63	34 (54.0)	29 (46.0)	0.305
No	24	10 (41.7)	14 (58.3)	
Multicentricity				
Yes	52	26 (50.0)	26 (50.0)	0.896
No	35	18 (51.4)	17 (48.6)	
Tumor size (cm)				
<2	43	16 (37.2)	27 (62.8)	0.014*
≥2	44	28 (63.6)	16 (36.4)	

*P < 0.05

### Overexpression of ATAD2 could partly impair si-NEAT1_2 induced inhibition of malignant behavior in PTC cells

**Fig. 5:**
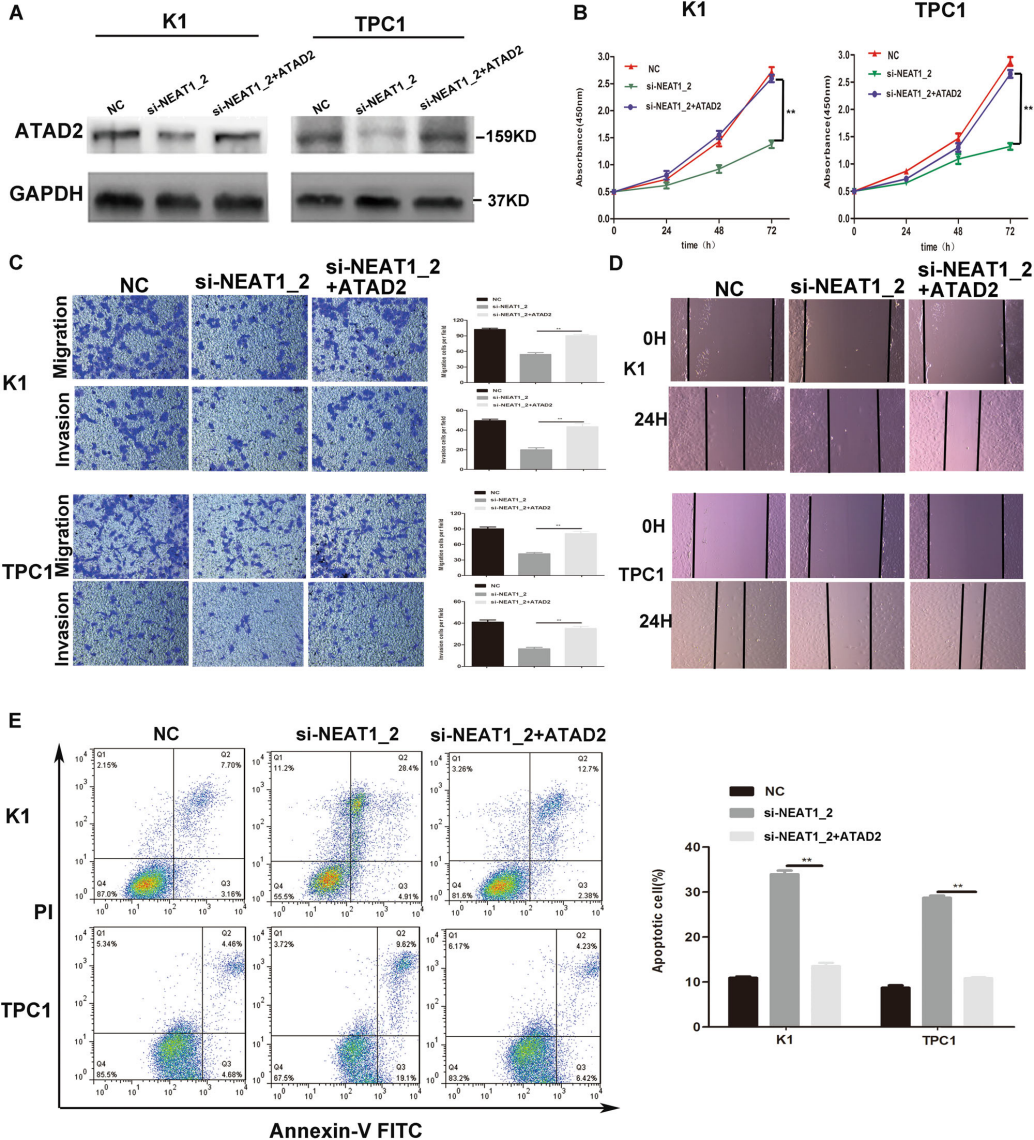
Overexpression of *ATAD2* could partly impair si-NEAT1_2 induced inhibition of malignant behavior in PTC cells. **a** Western blotting was used to analyze the level of ATAD2 in PTC cells transfected with si-NEAT1_2 or co-transfected pCDNA3.1-ATAD2 and si-NEAT1_2 or NC. **b** The CCK-8 assay was used to evaluate the cell growth after transfection with si-NEAT1_2 or co-transfected pCDNA3.1-ATAD2 and si-NEAT1_2 or NC in PTC cells. Data are presented as the mean ± S.D., analyzed using independent samples *t*-test. **P < 0.01 vs. the si-NEAT1_2 group. **c** Transwell assay analysis was used to evaluate the migration and invasion of PTC cells after transfection with si-NEAT1_2 or co-transfected pCDNA3.1-ATAD2 and si-NEAT1_2 or NC. Data are presented as the mean ± S.D., analyzed using independent samples *t*-test. **P < 0.01 vs. the si-NEAT1_2 group. **d** A wound healing assay was applied to analyze the migratory capacity of PTC cells after transfection with si-NEAT1_2 or co-transfected pCDNA3.1-ATAD2 and si-NEAT1_2 or NC. **e** Flow cytometry was used to evaluate the effect of apoptosis after transfection with si-NEAT1_2 or co-transfected pCDNA3.1-ATAD2 and si-NEAT1_2 or NC in PTC cell. Data are presented as the mean ± S.D., analyzed using independent samples *t*-test. **P < 0.01 vs. si-NEAT1_2 group

To confirm whether the tumor-suppressive effects of NEAT1_2 knockdown were mediated by ATAD2 in PTC cells, pCDNA3.1-ATAD2 and si-NEAT1_2 were co-transfected into two PTC cell lines. Relevant cell function assays were applied to assess the growth, migration, invasion, and apoptosis of the transfected PTC cells. As shown in Fig. 5a, western blotting was used to analyzed the level of ATAD2 in the different groups. The level of ATAD2 was significantly increased in the group co-transfected with pCDNA3.1-ATAD2 and si-NEAT1_2 compared with cells transfected with si-NEAT1_2 alone. Subsequent functional experiments showed that overexpression of ATAD2 could partly impair si-NEAT1_2 induced inhibition of growth (Fig. 5b), migration and invasion (Fig. 5c, d), and the promotion of apoptosis (Fig. 5e). Thus, the results demonstrated that overexpression of ATAD2 could rescue the inhibition of the malignant behavior of knocking down NEAT1_2. However, the results also indicated that overexpression of ATAD2 could only partly impair si-NEAT1_2-induced inhibition of the malignant behavior in PTC cells, which suggested that ATAD2 might just be one of the major downstream target genes regulated by NEAT1_2.

### Knockdown of NEAT1_2 inhibited ATAD2 expression by upregulating miR-106b-5p in PTC cells

**Fig. 6:**
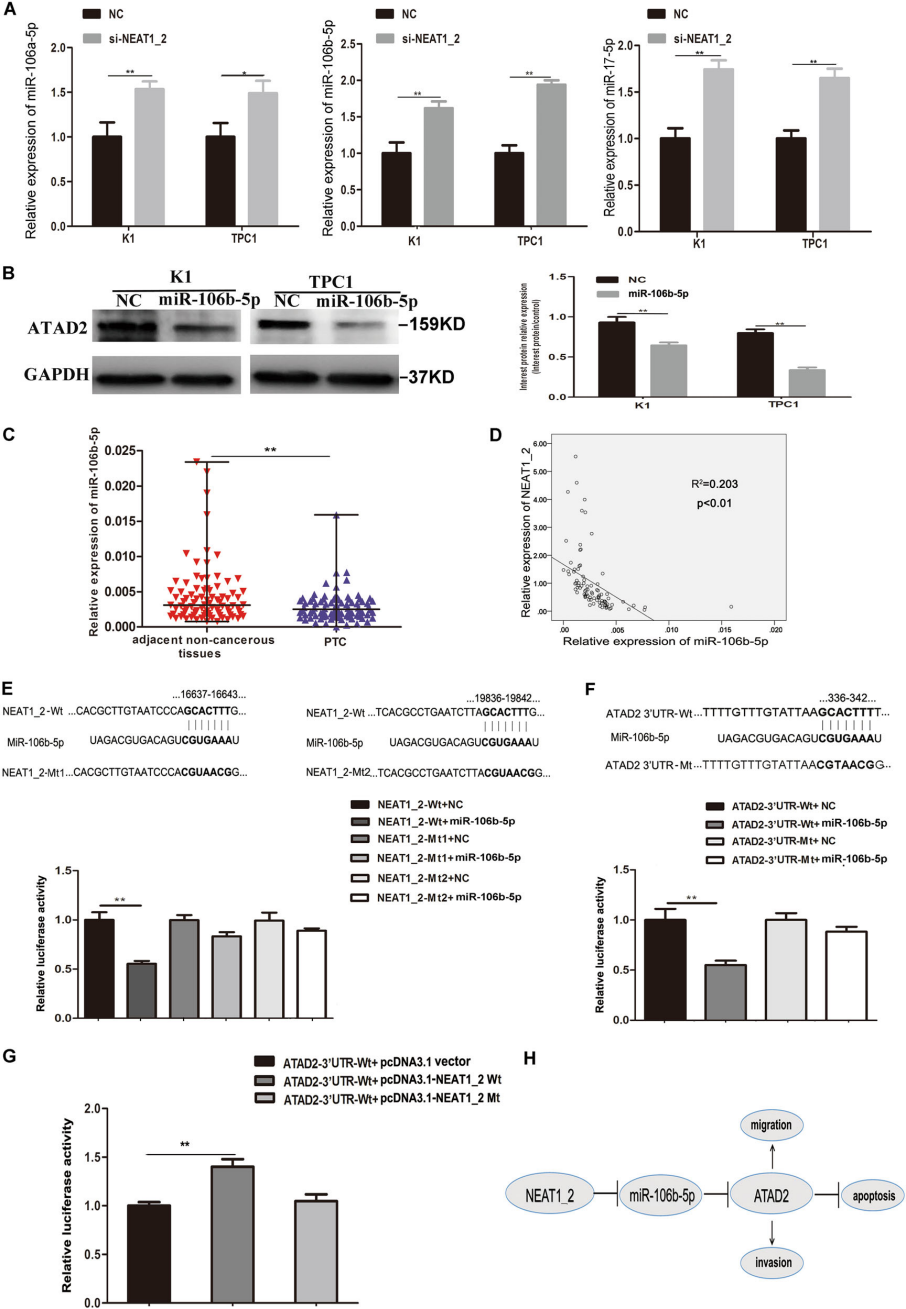
Knockdown of NEAT1_2 inhibited the ATAD2 expression by upregulating miR-106b-5p. **a** Relative expression levels of miR-106a-5p, miR-106b-5p, and miR-17-5p were detected by qRT-PCR in PTC cells transfected with si-NEAT1_2 or NC. Data are presented as the mean ± S.D., analyzed using independent samples *t*-test. *P < 0.05, **P < 0.01 vs. NC. **b** Protein expression of ATAD2 was detected by western blotting in PTC cells transfected with miR-106b-5p mimic or NC. Data are presented as the mean ± S.D., analyzed using independent samples *t*-test. **P < 0.01 vs. NC. **c** Relative miR-106b-5p levels were investigated in 87 adjacent non-cancerous tissues and in PTC tissues using qRT-PCR. A Wilcoxon signed-rank test was used to analyzed the differences between two groups; data were presented as the median with range. **P < 0.01 **d** Pearson’s correlation was performed to analyze the correlations between NEAT1_2 and miR-106b-5p expression in PTC tissues (*R*^2^ = 0.203, *P* < 0.01). **e** The predicted miR-106b-5p binding sites in NEAT1_2 (NEAT1_2-Wt) and the designed mutant sequence (NEAT1_2-Mt1, NEAT1_2-Mt2) are indicated. HEK 293T cells were transfected with NEAT1_2-Wt, NEAT1_2-Mt1, NEAT1_2-Mt2, and the indicated miRNAs, and then the luciferase reporter assay was conducted. Data are presented as the mean ± S.D., analyzed using independent samples *t*-test. **P < 0.01 vs. NEAT1_2-Wt + NC. **f** The predicted miR-106b-5p binding sites in the 3′-UTR region of ATAD2 (ATAD2-3′UTR-Wt) and the designed mutant sequence (*ATAD2*-3′UTR-Mt) are indicated. HEK 293T cells were transfected with *ATAD2*-3′UTR-Wt or *ATAD2*-3′UTR-Mt and the indicated miRNAs, and then the luciferase reporter assay was conducted. Data are presented as the mean ± S.D., analyzed using independent samples *t*-test. **P < 0.01 vs. *ATAD2*-3′UTR-Wt + NC. **g** *ATAD2*-3′UTR-Wt was respectively co-transfected with vectors pcDNA3.1, pcDNA3.1-NEAT1_2 Wt, and pcDNA3.1-NEAT1_2 Mt, and then the luciferase reporter assay was conducted. Data are presented as mean ± S.D., analyzed using independent samples *t*-test. **P < 0.01 vs. *ATAD2*-3′UTR-Wt + pcDNA3.1 vector. **h** Schematic of the proposed mechanism of NEAT1_2 in PTC. NEAT1_2 functions as a competing endogenous RNA to regulate *ATAD2* expression and promotes PTC progression by sponging miR-106b-5p

To further understand the mechanism of how downregulation of NEAT1_2 inhibited ATAD2 expression in PTC cells, we focused on whether NEAT1_2 could act as a ceRNA regulating ATAD2 by sponging miRNAs. Therefore, the bioinformatics analysis and target prediction tools miRWalk, TargetScan, and FINDTAR3 were applied to evaluate potential miRNAs that could not only target *ATAD2*, but also had binding sites for NEAT1_2. A total of 28 miRNAs were initially predicted to bind to both NEAT1_2 and the 3′ UTR of *ATAD2* (Supplementary Table 1). Then, qRT-PCR was used to confirm whether NEAT1_2 downregulation could significantly upregulate the expression of the 28 predicted miRNAs in K1 and TPC1 cells. We found that miR-106a-5p, miR-106b-5p, and miR-17-5p were significantly highly expressed in the NEAT1_2 knockdown group compared with their levels in the NC group (Fig. 6a). Next, we overexpressed miR-106a-5p, miR-106b-5p, and miR-17-5p, separately, and detected whether the three miRNAs could downregulate the expression of ATAD2 in K1 and TPC1 cells. We found that only miR-106b-5p could downregulate the expression of *ATAD2* (Fig. 6b). Neither miR-106a-5p nor miR-17-5p could downregulate the expression of *ATAD2* (Supplementary Figure 2). Thus, we found that miR-106b-5p targeted *ATAD2* and has two binding sites that interact with NEAT1_2. Moreover, we detected the relative expression of miR-106b-5p in 87 pairs of PTC and adjacent non-cancerous tissues. We found that miR-106b-5p showed significantly lower expression in PTC compared with that in adjacent non-cancerous tissues (Fig. 6c). The expression level of miR-106b-5p and NEAT1_2 were negatively correlated in 87 matched PTC tissues (Fig. 6d). In addition, dual-luciferase reporter assays were used to confirm whether miR-106b-5p could directly target ATAD2 and bind to NEAT1_2. We found that co-transfection of miR-106b-5p mimic and NEAT1_2 wild type (NEAT1_2-Wt) significantly reduced the luciferase activity, while miR-106b-5p mimic and NEAT1_2-mutated-type 1 (NEAT1_2-Mt1) or NEAT1_2-mutated-type 2 (NEAT1_2-Mt2) co-transfection failed to change the luciferase activity (Fig. 6e). Similarly, a dual-luciferase reporter system was carried out to determine whether *ATAD2* was a direct target of miR-106b-5p. We found that ectopic overexpression of miR-106b-5p significantly suppressed the luciferase activity of the wild-type *ATAD2*-3′UTR (*ATAD2*-3′ UTR-Wt), but failed to affect that from mutated-type *ATAD2*-3′UTR (*ATAD2*-3′ UTR-Mt) in HEK 293T cells (Fig. 6f). After confirming that miR-106b-5p could directly bind to both NEAT1_2 and the 3′ UTR of *ATAD2*, another dual-luciferase reporter assay was applied to further confirm whether NEAT1_2 could regulate *ATAD2* by interacting with miR-106b-5p. We found that pcDNA3.1-NEAT1_2 wild-type plasmid (pcDNA3.1-NEAT1_2 Wt) significantly elevated the luciferase activity of the *ATAD2*-3′UTR-Wt; however, pcDNA3.1-NEAT1_2 mutated-type plasmid (pcDNA3.1-NEAT1_2 Mt) did not affect the luciferase activity of *ATAD2*-3′ UTR-Wt (Fig. 6g). Taken together, these results demonstrated that NEAT1_2 could upregulate *ATAD2* expression by sponging miR-106b-5p.

## Discussion

In the human genome, only 1–2% of the entire genome encodes proteins, with evidence of at least 80% of the remaining genome being actively transcribed^24,25^. These non-protein coding portions of the genome produce a large variety of mostly regulatory RNAs that differ in their biogenesis, properties, and functions^26^. NEAT1 is an lncRNA that was first reported in a study to identify nuclear-enriched RNA transcripts in 2007. This lncRNA has been studied as the core structural component of paraspeckles. Paraspeckles have been reported to be involved in gene expression control by retaining mRNAs for editing in the nucleus. Studies of this relationship show that lncRNA NEAT1 may play an important role in the regulation of genes and consequent physiological and pathophysiological processes^10,27^. Recent studies indicated that NEAT1 is upregulated and plays a functional role in tumorigenesis. For example, it has been reported that NEAT1 can promote lung cancer progression through the miR-377-3p–E2F3 axis, regulate EMT through the miR-204/ZEB1 pathway in nasopharyngeal carcinoma, and serves as a downstream target of ERα to play a critical role in carcinogenesis of prostate cancer^12,20,28^. Moreover, NEAT1 expression is also a novel prognostic and diagnostic biomarker in gastric cancer, colorectal cancer, esophageal squamous cell carcinoma, and prostate cancer^12,29–31^. In our previous microarray assay, we found that NEAT1_2, a transcript of NEAT1, was significantly overexpressed in PTC tissues compared with adjacent non-cancerous tissues^17^. However, the expression, clinicopathological significance, and function of NEAT1_2 in PTC tissues and cell lines required further investigation.

Consistent with previous studies, the relative expression of NEAT1_2 was significantly increased in 87 matched PTC compared with that in adjacent non-cancerous tissues in this study. High relative expression of NEAT1_2 correlated with TNM stage and tumor size. We also detected the relative expression of NEAT1_2 in PTC cell lines. NEAT1_2 was overexpressed in K1 and TPC1 cells. Through cell function assays, we confirmed that the knockdown of NEAT1_2 inhibited growth, migration, and invasion, and promoted apoptosis, in PTC cells. Western blotting further detected the downstream effects on apoptosis-associated proteins Bcl-2, Bcl-xl and metastasis associated proteins/EMT-associated proteins, MMPs. These findings suggested that NEAT1_2 plays a role in the modulation of multiple oncogenic properties.

To investigate the downstream target proteins of NEAT1_2, we focused on detecting oncogenes associated with apoptosis and metastasis. We found that *ATAD2* was significantly downregulated in the si-NEAT1_2 group compared with the NC group, at both the mRNA and protein level. ATAD2, a member of the AAA + ATPase family of proteins, was identified by microarray analysis^32^. It contains both a bromodomain and an ATPase domain, and maps to chromosome 8q24 in a region that is frequently amplified in cancer^23^. The structure of ATAD2 suggests that it has functions related to genome regulation, including cell proliferation, differentiation, and apoptosis. Studies have revealed that ATAD2 is highly expressed and associated with proliferation and metastasis in several types of tumors, such as breast cancer, lung cancer, and hepatocellular carcinoma^33–38^. However, the expression and function of ATAD2 in PTC were unclear. Our study showed that *ATAD2* was overexpressed in 87 PTC tissues compared with adjacent non-cancerous tissues. Clinicopathological analysis indicated that the overexpression of *ATAD2* was associated with tumor size in PTC tissues. Further functional assays confirmed that *ATAD2* knockdown significantly affected cell growth, migration, invasion, and apoptosis in PTC cells, which suggested that ATAD2 plays an oncogenic role in PTC. NEAT1_2 might promote PTC metastasis and inhibit apoptosis by regulating the oncogene *ATAD2*.

Next, we adapted a “rescue” strategy to investigate the functional relevance of NEAT1_2 targeting of *ATAD2* in PTC cells. In the rescue assay, co-transfected pCDNA3.1-ATAD2 and si-NEAT1_2 could reverse the effects of si-NEAT1_2, including the inhibition of proliferation, migration, and invasion, and promotion of apoptosis. The rescue assays further confirmed that downregulation of NEAT1_2 inhibited malignant biological behavior by regulating *ATAD2*. However, we also found that overexpression of *ATAD2* could only partly impair si-NEAT1_2-induced inhibition of the malignant behavior in PTC cells, not all. ATAD2 might be just one of the major downstream target genes regulated by NEAT1_2. Identifying further target genes of NEAT1_2 in PTC will be a main focus of our future study.

Emerging evidence suggests that lncRNAs might function as competing endogenous RNAs (ceRNAs) or as a molecular sponge to modulate the activities of miRNAs^39,40^. NEAT1 has been reported acting as a ceRNA in the development of different cancers^18–20^. Therefore, we speculated that NEAT1_2 might also function as a ceRNA, regulating ATAD2 expression by sponging miRNAs in PTC. Bioinformatics analysis and target prediction tools identified miRNAs that could not only target *ATAD2*, but also have binding sites for NEAT1_2. Initially, 28 miRNAs were predicted to interact with both NEAT1_2 and the 3′ UTR of *ATAD2*. Among these 28 miRNAs, qRT-PCR indicated that miR-106a-5p, miR-106b-5p, and miR-17-5p were upregulated in PTC cells transfected with si-NEAT1_2 compared with the NC group. However, further western blotting results indicated that only miR-106b-5p could downregulate the level of *ATAD2* in PTC cells. Thus, miR-106-5p was speculated to be the miRNA that binds to both NEAT1_2 and the 3′ UTR of *ATAD2*. MiR-106b-5p, as a member of the miR-106b-25 cluster, has been confirmed to promote cancer cell proliferation and metastasis in prostate cancer, esophageal squamous cell carcinoma, and gastric cancer^41,42^. However, cumulative evidence indicates that the expression of miR-106b-5p is downregulated in thyroid cancer and breast cancer, where it might function as a tumor suppressor^43,44^. Furthermore, ectopic overexpression of miR-106b-5p downregulated C1orf24 expression, which induced apoptosis and suppressed invasion in thyroid cancer^43^. Consistently, we found that miR-106b-5p was significantly downregulated in 87 pairs of PTC tissues and matched normal tissues. The correlation analysis showed that the levels of miR-106b-5p and NEAT1_2 were negatively correlated. To further confirm that miR-106b-5p could bind directly to NEAT1_2 and the 3′ UTR of *ATAD2*, three dual-luciferase reporter assays were performed. The results suggested that miR-106b-5p was the crucial miRNA that binds to both NEAT1_2 and the 3′ UTR of *ATAD2*, and that NEAT1_2 could regulate the expression of *ATAD2* through sponging miR-106b-5p, which suggested the mechanism by which how NEAT1_2 promotes malignant biological behavior via modulation of *ATAD2*.

Based on the published studies, the functions of NEAT1 could be paraspeckle-dependent and paraspeckle-independent. In the case of paraspeckle-dependent activity, a recent study suggested that NEAT1 was capable of influencing gene transcription indirectly by sequestration of a transcriptional regulator in paraspeckles^45^. In the case of paraspeckle-independent activity, NEAT1 has been shown to localize to epigenetically active chromatin and may be an important activator of gene transcription^12,46^. In our study, miR-106b-5p was confirmed to bind directly to NEAT1_2. However, it remains unclear how miRNAs are recruited into paraspeckles to directly interact with NEAT1_2 and whether the recruitment process is paraspeckle-dependent or paraspeckle-independent. Recently, miRNAs and AGO2 proteins were found in the nucleus, and are able to degrade lncRNAs that are restricted to the nucleus^47,48^. For example, miRNA-9 can target the lncRNA MALAT1 for degradation in the nucleus^49^. Thus, we hypothesized that NEAT1_2 may function as a competing endogenous RNA to regulate *ATAD2* expression by sponging miR-106b-5p in an AGO2-dependent manner. In addition, study have reported that paraspeckle proteins possess many putative RNA-binding domains and may interact with NEAT1_2 or miRNAs.^11^ The paraspeckle proteins are not only restricted to the nucleus, but also are found in the cytoplasm^50^. They can move in and out of paraspeckles^50^. Thus, we speculated that NEAT1_2 may also interact with miR-106b-5p in a paraspeckle protein-dependent manner. This will be one of the main directions of our future research.

In conclusion, our study first identified the overexpression of the lncRNA NEAT1_2 in PTC tissues. NEAT1_2 expression was positively correlated with tumor size and TNM stage. Knockdown of NEAT1_2 inhibited the cell growth, migration, and invasion, and promoted apoptosis by functioning as a ceRNA to regulate *ATAD2* expression by sponging miR-106b-5p. Targeting NEAT1_2 could be a promising therapeutic strategy to treat PTC.

## Materials and methods

### Sample collection

Eight-seven pairs of PTC and adjacent non-cancerous tissues were obtained from patients undergoing surgery at the First Hospital of China Medical University between 2010 and 2015. All samples were immediately dissected, placed on ice, snap-frozen in liquid nitrogen, and stored at −80 °C until later use. Tissue samples from each patient consisted of two parts, PTC tissues and adjacent non-cancerous tissues, which were all confirmed by histopathological examination. In our study, because of the limitation of the size of the thyroid, the adjacent non-cancerous tissues were normal thyroid tissues located more than 2 cm away from the tumor margins on the same lobe or from the opposite lobe. The collected clinicopathological characteristics included age, gender, lymph node metastasis, extrathyroidal extension, TNM stage, multicentricity, and tumor size. None of the patients had received preoperative local or systemic treatment.

### Total RNA extraction and qRT-PCR

Total RNA was isolated from frozen specimens collected from tissue samples and cells using RNAiso (Takara, Dalian, China). The qRT-PCR was performed using SYBR Premix Ex Taq II (Takara) on a Light Cycler 480 system (Roche, USA). The thermocycling conditions included an initial denaturation step at 95 °C for 30 s, denaturation at 95 °C for 5 s, and annealing at 60 °C for 30 s for 40 cycles, dissociation stage at 95 °C for 60 s, 55 °C for 1 min, 95 °C for 30 s. The primer sequences were as follows: NEAT1_2 (sense): 5′-CTA GAG GCT CGC ATT GTG TG-3′; NEAT1_2 (antisense): 5′-GCC CAC ACG AAA CCT TAC AT-3′. ATAD2 (sense): 5′-GGG CTA GAA ACA TCG TTC AAA GT-3′; ATAD2 (antisense): 5′-GCA TGG ACT GGT TTA CAC CAC-3′. GAPDH (sense): 5′-GAA GGT GAA GGT CGG AGT C-3′; GAPDH (antisense): 5′-GAA GAT GGT GAT GGG ATT TC-3′. MiR-106b-5p (sense): 5′-TAA AGT GCT GAC AGT GCA GAT-3′. The antisense primer for the miRNA was provided by the 638313 Mir-X™ miRNA First-Strand Synthesis Kit (Takara). U6 (sense): 5′-CTC GCT TCG GCA GCA CA-3′; U6 (antisense): 5′-AAC GCT TCA CGA ATT TGC GT-3′. The 2^−ΔCT^ method (CT, cycle threshold) was used to calculate the relative expression levels. ΔCT indicated the difference in the CT value between the target and endogenous reference. GAPDH is one of the common references applied in relative quantification and has been used as an internal control in almost all NEAT1-related studies^12–20^. U6 is the most common used reference for miRNA expression^13,18–20^. In supplementary figure 3, the CT value of GAPDH and U6 showed no statistical difference between PTC tissues and adjacent non-cancerous tissues in this study (Wilcoxon signed-rank test, *P* > 0.05). Thus, we used GAPDH and U6 as reference genes to calculate lncRNA/mRNA expression and miRNA expression. In this study all RNA expression levels determined by qRT-PCR were relative quantities and not absolute quantities. Each PCR amplification was performed in triplicate to verify the stability and repeatability of the results.

### Cell culture

The BCPAP cell line was obtained from DSMZ (Braunschweig, Germany). The Nthy-ori 3-1 and K1 cell lines were purchased from the European Collection of Authenticated Cell Culture (ECACC, UK). IHH4 was obtained from the Health Science Research Resources Bank (Osaka, Japan). The TPC1 cell line was a gift from Professor Meiping Shen (Department of General Surgery, The First Affiliated Hospital of Nanjing Medical University, Nanjing, Jiangsu). The BCPAP cell line and Nthy-ori 3-1 cell line were cultured in Roswell Park Memorial Institute (RPMI)-1640 medium supplemented with 10% fetal bovine serum (FBS). K1 cells were maintained in Dulbecco’s modified eagle’s medium (DMEM):Ham’s F12:MCDB 105 (2:1:1) and 2 mM glutamine supplemented with 10% FBS. The IHH4 cells were maintained in a 1:1 mixture of RPMI-1640 and DMEM supplemented with 10% FBS. TPC1 cells were maintained in DMEM with 15% FBS. The short interfering RNA (siRNA) and negative control (NC) were purchased from Gene Pharma (Suzhou, China). The sequence was si-NEAT1_2 (sense): 5′-GGA GGA GUC AGG AGG AAU AUU-3′, si-ATAD2 (sense): 5′-GGA CCA AGA AGU CCU UAC UTT-3′, miR-106b mimic (sense) 5′-UAA AGU GCU GAC AGU GCA GAU-3′, miR-106a mimic (sense) 5′-AAA AGU GCU UAC AGU GCA GGU AG-3′, miR-17-5p mimic (sense) 5′-CAA AGU GCU UAC AGU GCA GGU AG-3′. The sequence of the negative control (NC) was 5′-UUC UCC GAA CGU GUC ACG UTT-3′. The PTC cells were transfected using Lipofectamine 2000 (Invitrogen) according to the manufacturer’s protocol.

### Cell growth assay

A Cell Counting Kit-8 (CCK-8; Dojindo, Kumamoto, Japan) was applied to assess cell growth. Approximately 3 × 10^3^ cells per well were seeded in 96-well plates in a final volume of 100 μl and then transfected with si-NEAT1_2, si-ATAD2, or NC. The absorbance was detected at 0, 24, 48, and 72 h after gene transfection. CCK-8 solution (10 μl) was added to each well and incubated for 3 h at 37 °C. Absorbance levels were measured at a wavelength of 450 nm. Experiments were performed in triplicate.

### Cell migration and invasion detection

After 24 h of transfection, the cell concentration of each group was adjusted to 5 × 10^4^ cells/ml with serum-free medium. The upper chamber of the Transwell chamber was filled with 200 μl of cell suspension, and the lower chamber was filled with 500 μl of medium supplemented with 10% FBS. Cells were incubated for 12 h for the migration assay and 36 h for the invasion assay, and the cells that had migrated through the filters were fixed with 4% paraformaldehyde and stained with 0.5% crystal violet. The invaded cells were then counted under an inverted microscope at ×100 magnification in five random visual fields. Experiments were performed in triplicate.

### Wound healing assay

After 24 h of transfection, 2 × 10^6^ cells per well were cultured in six-well plates until they reached 80% confluence, and scratches were created by scraping the cell layer across each well using a 200-μl pipette tip. Separated cells were washed out using phosphate-buffered saline (PBS). Wounded cultures were incubated in serum-free medium for 24 h, and images in three observation fields were randomly captured for each well. Experiments were performed in triplicate.

### Flow cytometry

Approximately 48 h after being transfected with si-NEAT1_2, si-ATAD2, or NC, PTC cells were harvested by trypsinization. The cells were then resuspended in PBS and the cells concentration was adjusted to 1 × 10^6^ cells/ml. After double staining with Annexin V-fluorescein isothiocyanate and propidium iodide according to the manufacturer’s instructions, cell apoptosis was determined using flow cytometry (BD Biosciences, USA) according to the manufacturer’s instructions. Experiments were performed in triplicate.

### Western blotting

The proteins in PTC cells were extracted using a Total Protein Extraction Kit (KeyGEN, Nanjing, China). The protein extracts (20–30 μg) were separated on 10% sodium dodecyl sulfate polyacrylamide gel electrophoresis gels and then electrophoretically transferred to polyvinylidene difluoride membranes (Millipore, USA). After blocking with 5% non-fat milk for 2 h, the membranes were incubated with primary antibodies recognizing E-cadherin, N-cadherin, Vimentin, MMP9, MMP2, Bcl-2, Bcl-xl (1:2000 dilution; Abcam, USA), ATAD2 (1:1000 dilution; Abcam, USA), and GAPDH (1:1000 dilution; ZSGB-Bio, China) overnight at 4 °C. Protein bands were visualized by chemiluminescence (Thermo, USA) after incubation with secondary antibodies (1:10,000 dilution; Cell Signaling Technology, USA).

### Luciferase assay

The fragment from NEAT1_2 containing the putative binding sites for miR-106b-5p was amplified by PCR and cloned in the firefly luciferase expression vector pMIR-REPORT (Obio Technology, China) and named as NEAT1_2-Wt. To mutate the putative binding sites for miR-106b-5p in NEAT1_2, the sequence of putative binding site was replaced as indicated and was named as NEAT1_2-Mt1 and NEAT1_2-Mt2. HEK 293T cells were seeded into 96-well plates the day before transfection, and transfected with the pMIR-REPORT-NEAT1_2-lncRNA-Wt, pMIR-REPORT-NEAT1_2-lncRNA-Mt1, and pMIR-REPORT-NEAT1_2-lncRNA-Mt2 reporter vector, together with the Renilla luciferase-expressing vector pRL-TK (Promega, Madison, WI, USA) and miR-106b-5p mimic or NC using Lipofectamine 2000 (Invitrogen). Similarly, the *ATAD2*-3′UTR-Wt and *ATAD2*-3′UTR-Mt containing the putative binding site of miR-106b-5p were established and cloned into the Firefly luciferase expression vector pMIR-REPORT (Obio Technology, China). HEK 293T cells were seeded into 96-well plates the day before transfection, and transfected with either the pMIR-REPORT-*ATAD2*-3′UTR-Wt or the pMIR-REPORT-*ATAD2*-3′UTR-Mt reporter vector, together with the Renilla luciferase expression vector pRL-TK (Promega) and miR-106b-5p mimic or NC using Lipofectamine 2000 (Invitrogen). After 48 h, the cells were harvested, and firefly and Renilla luciferase activities were measured using the dual-luciferase reporter assay system (Promega).

To further confirm that NEAT1_2 could regulate *ATAD2* through interacting with miR-106b-5p, we constructed a pcDNA3.1-NEAT1_2 Wt containing both binding sites for miR-106b-5p and the pcDNA3.1-NEAT1_2 Mt containing mutated binding sites for miR-106b-5p. Briefly, we co-transfected the pMIR-REPORT-*ATAD2*-3′UTR-Wt reporter vector together with the Renilla luciferase-expressing vector pRL-TK (Promega) and pcDNA3.1 vector, pcDNA3.1-NEAT1_2 Wt, or pcDNA3.1-NEAT1_2 Mt using Lipofectamine 2000 (Invitrogen). After 48 h, cells were harvested, and firefly and Renilla luciferase activities were measured using the dual-luciferase reporter assay system (Promega).

### Statistical analysis

SPSS 13.0 software (SPSS, USA) was used for statistical analyses. The Wilcoxon signed-rank test was used to analyze the different relative expressions of NEAT1_2, *ATAD2*, and miR-106b-5p in PTC tissues and adjacent non-cancerous tissues. The chi-square test was applied to exam the relationship between NEAT1_2 or ATAD2 expression and clinicopathological characteristics. A two-independent sample *t*-test assuming a single (equal) variance per test was used to perform comparisons between two independent groups. Results were considered statistically significant at *P*-values <0.05.

## Electronic supplementary material

Supplementary Information
