# Supplementary material for: NEAT1_2 functions as a competing endogenous RNA to regulate ATAD2 expression by sponging microRNA-106b-5p in papillary thyroid cancer
Source: Cell Death Dis. 2018 Mar 7;9(3):380. doi: 10.1038/s41419-018-0418-z (PMC5841310; doi:10.1038/s41419-018-0418-z)
Supplement: Supplementary file 1 — Supplementary Information [file 41419_2018_418_MOESM1_ESM.doc]

| 28 predicted miRNAs both binding to NEAT1_2 and 3′ UTR of ATAD2 | qRT-PCR Primer (sense) |
| --- | --- |
| hsa-miR-186-5p | CAAAGAAUUCUCCUUUUGGGCU |
| hsa-miR-493-5p | UUGUACAUGGUAGGCUUUCAUU |
| hsa-miR-520f-3p | AAGTGCTTCCTTTTAGAGGGTT |
| hsa-miR-302c-3p | UUGUACAUGGUAGGCUUUCAUU |
| hsa-miR-520b | AAAGTGCTTCCTTTTAGAGGG |
| hsa-miR-373-3p | GAAGTGCTTCGATTTTGGGGTGT |
| hsa-miR-302d-3p | TAAGTGCTTCCATGTTTGAGTGT |
| hsa-miR-520c-3p | AAAGTGCTTCCTTTTAGAGGGT |
| hsa-miR-302e | TAAGTGCTTCCATGCTT |
| hsa-miR-302a-3p | TAAGTGCTTCCATGTTTTGGTGA |
| hsa-miR-302b-3p | TAAGTGCTTCCATGTTTTAGTAG |
| hsa-miR-302c-3p | UAAGUGCUUCCAUGUUUCAGUGG |
| hsa-miR-372-3p | AAAGTGCTGCGACATTTGAGCGT |
| hsa-miR-520a-3p | AAAGTGCTTCCCTTTGGACTGT |
| hsa-miR-520d-3p | AAAGTGCTTCTCTTTGGTGGGT |
| hsa-miR-20b-5p | CAAAGTGCTCATAGTGCAGGTAG |
| hsa-miR-526b-3p | GAAAGTGCTTCCTTTTAGAGGC |
| hsa-miR-106a-5p | AAAAGTGCTTACAGTGCAGGTAG |
| hsa-miR-17-5p | CAAAGTGCTTACAGTGCAGGTAG |
| hsa-miR-106b-5p | TAAAGTGCTGACAGTGCAGAT |
| hsa-miR-20a-5p | TAAAGTGCTTATAGTGCAGGTAG |
| hsa-miR-519d-3p | CAAAGTGCCTCCCTTTAGAGTG |
| hsa-miR-93-5p | CAAAGTGCTGTTCGTGCAGGTAG |
| hsa-miR-219a-2-3p | AGAAUUGUGGCUGGACAUCUGU |
| hsa-miR-374c-5p | AUAAUACAACCUGCUAAGUGCU |
| hsa-miR-203a-3p | GUGAAAUGUUUAGGACCACUAG |
| hsa-miR-374b-5p | AUAUAAUACAACCUGCUAAGUG |
| hsa-miR-374a-5p | UUAUAAUACAACCUGAUAAGUG |

**Supplement materials**

**Table 1.** **28 predicted miRNAs both binding to NEAT1_2 and 3′ UTR of ATAD2.**


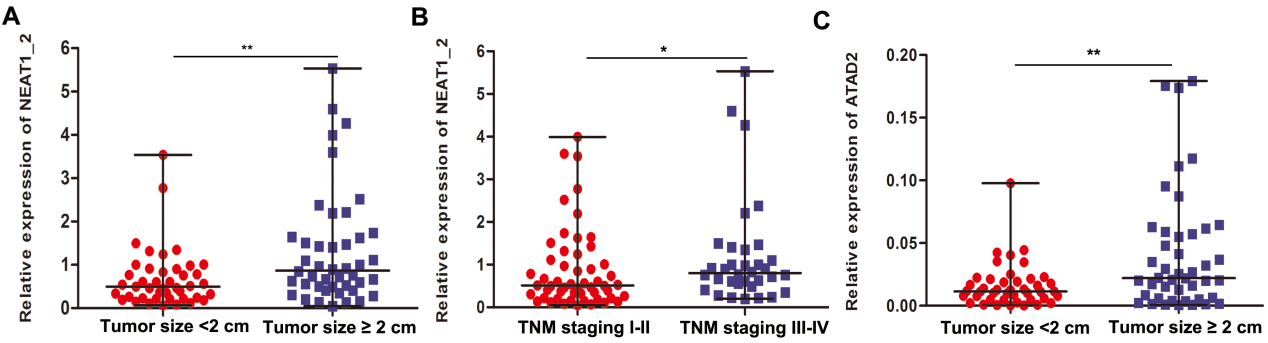


**Figure 1** NEAT1_2 or ATAD2 expression correlate with PTC clinical pathological features. **A** NEAT1_2 expression in the tumor <2cm (n=43) and tumor ≥2cm (n=44). Mann-Whitney U test was used to analyzed the differences between the two groups, data are presented as the median with range. **P < 0.01. **B** NEAT1_2 expression in the TNM staging I-II (n=53) and TNM staging III-IV (n=34). Mann-Whitney U test was used to analyzed the differences between the two groups, data are presented as the median with range. *P < 0.05. **C** ATAD2 expression in the tumor <2cm (n=43) and tumor ≥2cm (n=44). Mann-Whitney U test was used to analyzed the differences between the two groups, data are presented as the median with range. **P < 0.01.


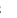


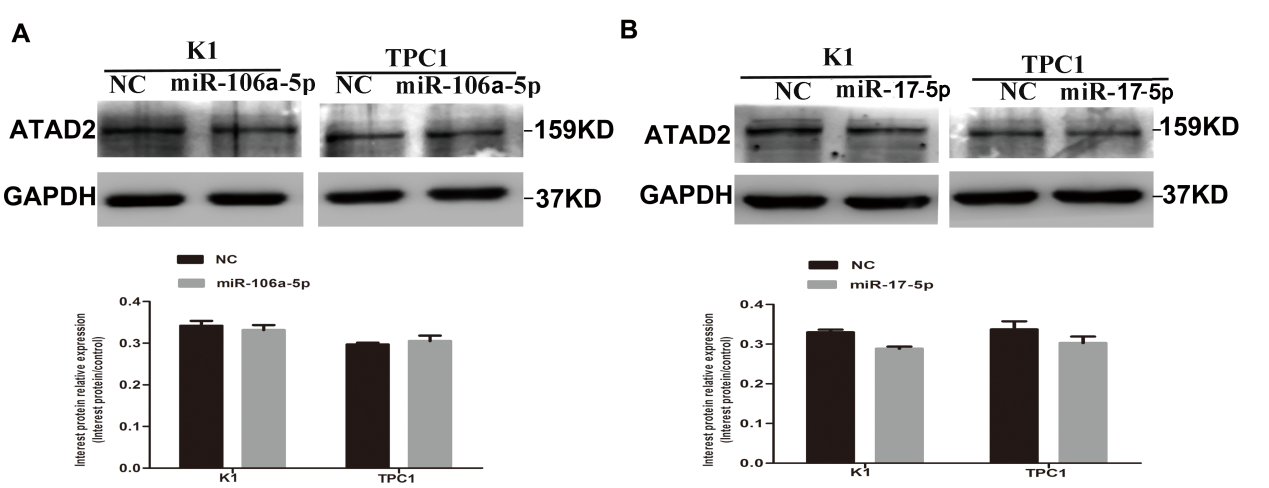


**Figure 2** The ATAD2 protein expression level. **A** Protein expression of ATAD2 was detected by western blotting in PTC cells transfection with miR-106a-5p mimic or NC. Data are presented as mean±S.D., analyzed using independent samples t-test. **B** Protein expression of ATAD2 was detected by western blotting in PTC cell transfection with miR-17-5p mimic or NC. Data are presented as mean±S.D., analyzed using independent samples t-test.


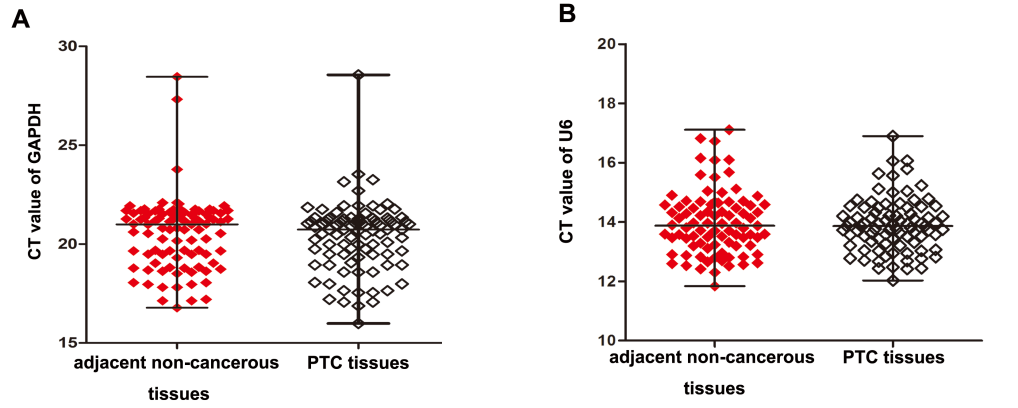


**Figure 3** The CT value of reference gene in 87 adjacent non-cancerous tissues and PTC tissues. **A** The CT value of GAPDH in 87 adjacent non-cancerous tissues and PTC tissues **B** The CT value of U6 in 87 adjacent non-cancerous tissues and PTC tissues. Wilcoxon signed-rank test was used to analyzed the differences between the two groups, data are presented as the median with range. No statistical difference was found in CT value of GAPDH and U6 between adjacent non-cancerous tissues and PTC tissues (p>0.05).
